# hERG1 channels are overexpressed in glioblastoma multiforme and modulate VEGF secretion in glioblastoma cell lines

**DOI:** 10.1038/sj.bjc.6602775

**Published:** 2005-09-20

**Authors:** A Masi, A Becchetti, R Restano-Cassulini, S Polvani, G Hofmann, A M Buccoliero, M Paglierani, B Pollo, G L Taddei, P Gallina, N Di Lorenzo, S Franceschetti, E Wanke, A Arcangeli

**Affiliations:** 1 Department of Experimental Pathology and Oncology, University of Firenze, Viale GB Morgagni, 50, 50134 Firenze, Italy; 2 Department of Biotechnology and Biosciences, University of Milano Bicocca, Piazza della Scienza, 2, 20126 Milano, Italy; 3 Department of Human Pathology and Oncology, University of Firenze, Viale GB Morgagni, 88, 50134 Firenze, Italy; 4 Istituto Neurologico Besta, Via Celoria, 11, 20133 Milano, Italy; 5 Department of Neurosurgery, University of Firenze, Largo P Palagi 1, 50139, Firenze, Italy

**Keywords:** brain tumours, angiogenesis, KCNH2, Kv 11.1, KCNH3, Kv 12.2

## Abstract

Recent studies have led to considerable advancement in our understanding of the molecular mechanisms that underlie the relentless cell growth and invasiveness of human gliomas. Partial understanding of these mechanisms has (1) improved the classification for gliomas, by identifying prognostic subgroups, and (2) pointed to novel potential therapeutic targets. Some classes of ion channels have turned out to be involved in the pathogenesis and malignancy of gliomas. We studied the expression and properties of K^+^ channels in primary cultures obtained from surgical specimens: human *ether a gò-gò* related (hERG)1 voltage-dependent K^+^ channels, which have been found to be overexpressed in various human cancers, and human *ether a gò-gò-like* 2 channels, that share many of hERG1's biophysical features. The expression pattern of these two channels was compared to that of the classical inward rectifying K^+^ channels, IRK, that are widely expressed in astrocytic cells and classically considered a marker of astrocytic differentiation. In our study, hERG1 was found to be specifically overexpressed in high-grade astrocytomas, that is, glioblastoma multiforme (GBM). In addition, we present evidence that, in GBM cell lines, hERG1 channel activity actively contributes to malignancy by promoting vascular endothelial growth factor secretion, thus stimulating the neoangiogenesis typical of high-grade gliomas. Our data provide important confirmation for studies proposing the hERG1 channel as a molecular marker of tumour progression and a possible target for novel anticancer therapies.

Gliomas are the most common brain tumours. Based on morphology and histological features, they are classified as astrocytomas (A), oligodendrogliomas (OD), mixed oligoastrocytomas (OA) or ependimomas (Ep) (Maher *et al*, 2001). Each of the above classes can be further subdivided according to the grading criteria outlined by the Word Health Organization (WHO; Kleihues and Cavenee, 2000). Tumour grade is usually considered the primary determinant of clinical outcome, although histogenesis contributes as well. For example, among high-grade gliomas, anaplastic ODs have a more favourable prognosis than glioblastoma multiforme (GBM) (i.e. WHO grade IV A). Moreover, primary (or *de novo*) GBM, more frequently affecting old patients, have a worse prognosis than secondary GBM, typically arising in young patients as a recurrence of a previous, lower-grade lesion. Therefore, a classification merely based on histopathological features is inadequate. Creation of a new classification of gliomas, with prognostic value and based on molecular properties, has recently been proposed (Huang *et al*, 2000; Louis *et al*, 2001; Rickman *et al*, 2001; Godard *et al*, 2003; Nutt *et al*, 2003). Recent studies have shed some light on the molecular mechanisms that underlie gliomagenesis (Maher *et al*, 2001) and regulate glioma growth and invasiveness. Particular attention has been given to neoangiogenesis, typical of high-grade GBM (Godard *et al*, 2003; Kitange *et al*, 2003). According to these studies, primary GBMs can be distinguished from secondary GBMs by their expression of genes involved in neoangiogenesis. This kind of understanding of molecular mechanisms is assuming greater importance in the design of treatments tailored specifically to each tumour subset. For example, a promising approach to treatment of gliomas is the targeting of molecules involved in neoangiogenesis, such as the vascular endothelial growth factor (VEGF) and its receptors.

An intriguing aspect of the biology of malignant gliomas is that it is the same ion transport mechanisms that normally control homeostatic processes such as the regulation of cell volume (Bostel *et al*, 2002), proliferation and differentiation (Pancrazio *et al*, 1999), that often become part of the pathogenesis and malignancy of these tumours. The expression profile of ion channels is known to be profoundly altered in glial tumour cells (Bordey and Sontheimer, 1998; Bubien *et al*, 1999; Ranson and Sontheimer, 2001; Olsen and Sontheimer, 2004). In some contexts, these alterations have been shown to trigger relentless growth (reviewed in Arcangeli and Becchetti, 2006) and invasiveness (Soroceanu *et al*, 1999; Ishiuchi *et al*, 2002; Olsen *et al*, 2003). In other cases, the expression profile of specific ion channels may change as a result of a local reaction to a pathological state, such as trauma, infection or the tumour itself (Saadoun *et al*, 2003). A recent report indicates that, when expressed at the blood–brain barrier at the tumour site, potassium channels can even restrict the delivery of anticancer drugs (Ningaraj *et al*, 2003). On the whole, it appears that ion channels/transporters, normally functioning to control ion homeostasis in a healthy brain, may contribute to the establishment of the malignant phenotype of brain tumours. This evidence points to ion channels as novel molecular markers for malignant gliomas and as new candidate targets for therapy (Conti, 2004; Arcangeli, 2005).

The human *ether a gò-gò* related (hERG1) channels (KCNH2 or Kv 11.1, according to the most recent nomenclature) are voltage-dependent K^+^ channels that are overexpressed in human endometrial adenocarcinoma (Cherubini *et al*, 2000), myeloid leukaemia (Pillozzi *et al*, 2002) and colorectal cancer (Lastraioli *et al*, 2004). In addition, they are often overexpressed in neoplastic cell lines of different origin. Interestingly, in primary cultures from leukaemia blasts, and in many cell lines, hERG1 inhibition tends to block cell proliferation (reviewed in Arcangeli, 2005; Arcangeli and Becchetti, 2005).

To build on these studies, we examined the expression and properties of hERG1 channels and the functionally related human *ether a gò-gò-like* (hELK)2 channels (Miyake *et al*, 1999; Becchetti *et al*, 2002; KCNH3 or Kv 12.2). The expression pattern of these two channels was compared to that of the classical inward rectifying (IRK) K^+^ channels (KCNJ2 or Kir 2.1), widely expressed in astrocytic cells, and whose plasma membrane expression is downregulated in gliomas (Olsen and Sontheimer, 2004).

It emerged that hERG1 channels are specifically overexpressed in high-grade A, particularly in primary GBM. This finding prompted us to investigate how hERG1 expression may represent a selective advantage to glial tumour cells. Due to the significance of angiogenic factor secretion in conferring high malignancy to gliomas, and since several studies indicate a role for hERG currents (*I*_hERG_) in neuroendocrine secretion (Rosati *et al*, 2000; Bauer *et al*, 2003; Gullo *et al*, 2003), we sought a functional link between hERG1 activity and the secretion of angiogenic factors, such as VEGF.

## MATERIALS AND METHODS

### Tissue collection

Surgical specimens were obtained from the Istituto Neurologico Besta of Milano and the Department of Neurosurgery of the University of Firenze.

### Histological examinations

The histological study on tumour samples was performed at the Neuropathology Section of the Istituto Neurologico Besta, Milano, and at the Department of Human Pathology and Oncology, University of Firenze, Firenze. Specimens were fixed in different solutions (Carnoy's solution was routinely used for samples processed in Milano, while 3.7% formalin solution was routinely used for samples processed in Firenze). All samples were embedded in paraffin. Histopathological evaluation was performed on haematoxylin–eosin-stained sections. Pathologists (BP, GLT, AMB) determined the histological diagnosis using standard criteria (Buccoliero *et al*, 2002).

### Primary cell cultures

Surgical fragments of brain tumours were collected under sterile conditions and kept in Dulbecco's modified Eagle Medium:F12 (DMEM : F12) (1 : 1) plus 1% penicillin–streptomycin/fungizone (Hyclone Laboratories, Logan UT), until dissociation. Samples were first thoroughly minced and then enzymatically dissociated by a 6 h digestion at 37°C with collagenase II (Roche Diagnostic, Mannheim, Germany) 0.05 mg ml^−1^ in DMEM : F12. Dissociated cells were centrifuged and then resuspended in complete medium (DMEM : F12, medium supplemented with antibiotics and 10% foetal calf serum (FCS, Hyclone)) and seeded into either 35 mm Petri dishes (Corning-Costar, Corning, NY) for patch-clamp experiments, or into 60 mm Petri dishes for molecular biology experiments, or in chamber slides (BD Falcon, Franklin Lakes, NJ) for immunocytochemical detection of glial fibrillary acidic protein (GFAP)). After a 24 h incubation, cell cultures were rinsed with sterile PBS, and fresh medium was added. Experiments were normally performed on cells cultured for less than 5 days, since glioma cells tend to lose glial and acquire a mesenchymal phenotype when subjected to long-term culture (McKeever *et al*, 1991).

### Reverse transcription (RT) and polymerase chain reaction (PCR) amplification

Cells were rinsed twice in cold sterile PBS and lysed in a guanidinium thiocyanate buffer. Samples were then extracted by the phenol/chloroform method. Total RNA was quantified by spectrophotometric analysis and checked for purity and quality by running a small aliquot on a 1% agarose gel. 1–2 *μ*g were reverse transcribed with Superscript II (Invitrogen, Carlsbad, CA), using 250 *μ*M random hexamers, in a 20 *μ*l reaction mix. RNAse inhibitor (1 U) (Roche) was used to avoid RNA degradation. The reaction was carried out following manufacturer's instructions. Total cDNA was used as a template for PCR amplification with Herculase DNApol (Stratagene, Cedar Creek, TX) and each of the primer pairs reported below. As a quality control on cDNA, PCR amplification of the messenger relative to the housekeeping gene *gapdh* was performed using the following primer pair:

Fw: 5′-AACAGCCTCAAGATCATCAGCAA-3′

Rev: 5′-CAGTCTGGGTGGCAGTGAT-3′

(NG 003027, nucleotides 457–564)

Samples positive to *gapdh* amplification were further analysed using the following primers:

*hERG1* fw: 5′-TCCAGCGGCTGTACTCGGGC-3′

*hERG1* rev: 5′-TGGACCAGAAGTGGTCGGAGAACTC-3′

(U04270, nucleotides 2171–2746).

*helk2* fw: 5′-CTGCCCTGCGGGGCACTGTCTG-3′

*helk2* rev: 5′-AGATCTGGGGGCACATTCCATG-3′

(NM012284 nucleotides 1802–2516).

*Kir 2.1* fw: 5′-GTGATTGCCATGAGAGACGGC-3′

*Kir 2.1* rev: 5′-TCTTCCTCCTTTGCTTGTGAGG-3′

(U 12507, nucleotides 923–1488).

*hEAG* fw: 5′-CGCATGAACTACCTGAAGACG-3′

*hEAG* rev: 5′-TCTGTGGATGGGGCGATGTTC-3′

(NM 002238, nucleotides 1032–1510 and NM 172362, nucleotides 1032–1591).

PCR protocols consisted in a 2 min initial activation step at 95°C, followed by 35 cycles of amplification. Each cycle included 30 s at 94°C, 1 min at the specific annealing temperature (56°C for *herg1*, 64°C for *helk2* and 60°C for *Kir 2.1*, *gapdh* and *hEAG*) and 1 min extension at 68°C. Total human brain RNA (Ambion, Austin, TX) was used as a control for expression of all the above transcripts. As a control for RNA purity, mock reverse-transcription mixes (where RT was omitted) were PCR amplified along with samples: no unspecific bands, due to genomic DNA contamination, have ever been observed.

For semiquantitative PCR of VEGF transcripts, RNA was extracted from U138 cells and reverse-transcribed as above. The following primers were used, according to Meister *et al* (1999):

*vegf* fw: 5′-CGAAGTGGTGAAGTTCATGGATG-3′

*vegf* rev: 5′-TTCTGTATCAGTCTTTCCTGGTGAG-3′

These primers span the insertion/deletion site of human VEGF_165_ and amplification of the transcripts encoding the 121-, 165- and 198-amino-acid isoforms yields PCR products of 403, 535 and 607 bp. Conditions were the same as those applied for amplification of channel-encoding genes, except for a 50°C annealing temperature. All primers were tested over a range of cycle numbers to assure that amplification was still in a logarithmic phase of visible product increase. For semiquantitative analysis of the *vegf* transcript 20, 25, 30 and 35 cycles were performed, while for *gapdh* 15, 20 and 25 cycles were performed. A control amplification on RNA extracted from HUVEC cells was included.

### Patch-clamp recordings

Whole-cell currents were recorded from primary cell cultures obtained from brain tumour specimens, within 3–4 days. Cells were voltage-clamped at room temperature with an Axopatch 200B amplifier (Axon Instruments, Foster City, CA, USA). The cell capacitance and series resistance were compensated (75–85%) before each stimulation protocol was run. Pipette resistances were 2–5 MΩ. Currents were low-pass filtered at 2 kHz and digitised on-line at 10 kHz with pClamp (Axon Instruments) hardware and software. Data were subsequently analysed off-line with pClamp and Origin (Microcal Inc., Northhampton, MA, USA) software.

Extracellular solutions were delivered through a nine-hole (0.6 mm), remote-controlled linear positioner placed near the cell under study. The standard extracellular solution (SES) contained (mM): NaCl 130, KCl 5, CaCl_2_ 2, MgCl_2_ 2, Hepes-NaOH 10, glucose 5, pH 7.4. The high K^+^ solution had the same composition, except that the NaCl and KCl concentrations were 95 and 40 mM, respectively. Pipette contained (in mM): K^+^-aspartate 130, NaCl 10, MgCl_2_ 2, CaCl_2_ 1.3, EGTA-KOH 10, Hepes-KOH 10, ATP (Mg^2+^ salt) 1, pH 7.3. The calculated pCa was 7. The pharmacological compounds were added to the external solution. The antiarrhythmic drug Way 123,398 (WAY, kindly gifted by Dr W Spinelli, Wyeth-Ayerst Research, Princeton, NJ, USA) was used at a concentration of 1 *μ*M. All salts and the other drugs were purchased from Sigma. To measure inward K^+^ currents at practical test potentials, we artificially decreased the Nernst potential for K^+^ (*E*_K_) by exposing cells under investigation to the high-K^+^ external solution. Subsequently, we applied voltage-clamp steps from −140 to +20 mV, preceded by a 10 s conditioning step at 0 mV. The long duration of the conditioning step was required by the slow kinetics of hERG1 channels (Schönherr *et al*, 1999) and is indifferent for IRK. The holding potential was −60 mV.

The *I*_hERG_ activation curve was obtained from peak tail currents at −120 mV, elicited after 3 s conditioning steps from −60 to + 40 mV. The resting potential (*V*_rest_) of U138 and A172 glioma cell lines was measured in SES, in open-circuit mode.

The effect of WAY on *I*_hERG_ in different experimental conditions was tested on HEK-hERG1 stably transfected cells (Lastraioli *et al*, 2004). Whole-cell currents were studied by using a two-step protocol (Hofmann *et al*, 2001). Conditioning steps at 0 and −70 mV (lasting 10 s) were followed by an 80 ms step, at −120 mV. Stock solutions of WAY were prepared in distilled water at the concentration of 5 mM. The inhibitor was applied to the cells (1) immediately after diluting in SES; (2) after diluting in the same culture medium used for VEGF secretion experiments (CM; see below), and incubating for 15 min, at 37°C, in 5% CO_2_; (3) after diluting in CM and incubating for 24 h, at 37°C, in 5% CO_2_ in the culture plates containing the cells (CM 24 h). For each condition, two different WAY concentrations (1 and 40 *μ*M) were tested. The effect of WAY is reported as percentage inhibition, calculated as 1−(*I*_hERG_WAY/*I*_hERG_SES) × 100, where *I*_hERG_WAY and *I*_hERG_SES are the *I*_hERG_ values measured in the presence or in the absence of WAY (i.e. in SES), respectively.

### Immunohistochemistry (IHC)

For hERG1 detection, an anti-hERG1 antibody produced in our laboratory (now available from Immunological Sciences, Roma) was used at 1 : 200 dilution. Antigen retrieval and staining conditions were the same as those reported in Lastraioli *et al* (2004). hERG1 immunochemical detection was carried out using commercially available kits (PicTure Plus kit, Zymed Laboratories, CA, USA). For GFAP and NF IHC, we used the method previously described in Buccoliero *et al* (2002). In this case, immunodetection was carried out using standard streptavidin–biotin technique and performed on NEXES automated immunostainer. Antibodies were the following: mouse anti-GFAP monoclonal (ZCG29) prediluted, Zymed Laboratories, CA, USA; mouse anti-NF polyclonal (clones: FNP, DA2, RMd020.11), 1 : 30 dilution, Zymed Laboratories, CA, USA. Microwave antigen enhancement was utiliSed for anti-GFAP and -NF antibodies. For all the above antibodies, diaminobenzidine (DAB, Zymed Laboratories, CA, USA) was used, so that antigen-expressing cells were distinguished from the negative ones because of the presence of a brown to black precipitate.

### Cell culture and VEGF detection

U138 and A172 cells (kindly provided by Dr A Colombatti, Centro di Riferimento Oncologico, Aviano) were routinely cultured in DMEM high glucose plus 10% FCS (Defined, Hyclone). The detection of hERG1 channel by protein extraction and Western blotting was performed as reported in Lastraioli *et al* (2004). For testing VEGF secretion, cells were seeded into 24-well cell clusters at 3 × 10^5^ cells per well in Opti-Mem (Invitrogen). After 48 h, cell culture medium was changed and WAY or E4031 was added to the final concentration reported in the legend to Figure 6. After a 24 h incubation, the medium was collected and used for measurement of secreted VEGF (DuoSet ELISA Development System, R&D Systems, Wiesbaden, Germany), or bFGF (Quantikine FGF basic Immunoassay, R&D Systems, Wiesbaden, Germany). Cells were subsequently detached and counted.

### Statistics

Data are given as average values±standard errors of the mean (s.e.m.), with the number of cases reported within parentheses. When necessary, a Student's *t*-test was applied to test for significance.

## RESULTS AND DISCUSSION

A total of 41 cases of glioma were enrolled in the present study. Their clinical–pathological characteristics are reported in Table 1. Among them, 19.5% were Grade I–III As, 63.4% were GBMs, 12.2% were ODs or OAs, and 4.9% were Eps. As controls, we included five samples of normal brain tissue: three of them were obtained from patients operated on for untreatable epilepsy, two were fragments of normal brain tissue external to the tumour mass, from glioma patients. Following excision, tumour fragments were immediately dissociated and cultured as described in Materials and Methods. The glial identity of our cultures was assessed by immunofluorescence staining for GFAP, within 2 days of seeding. Only primary cultures showing homogeneous staining for GFAP were considered.

### K^+^ currents in primary human gliomas

**Figure 1:**
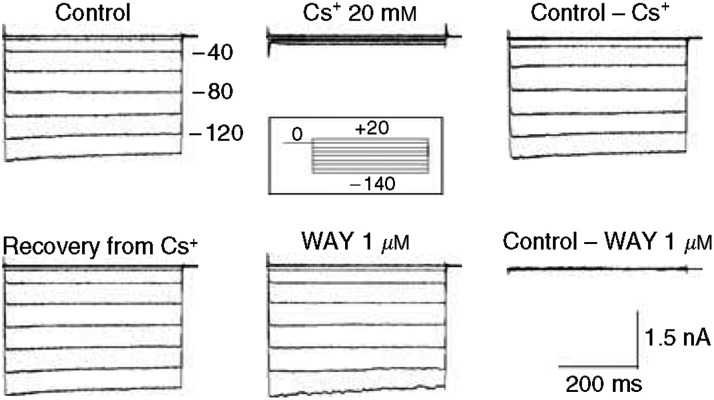
*I*_IRK_ currents in non-neoplastic tissue. Whole-cell current traces elicited by the stimulation protocol shown in the inset, in the absence (control) or presence of the indicated K^+^ channel inhibitors. The extracellular solution contained 40 mM K^+^, giving an *E*_k_ around −30 mV. Cells were conditioned at 0 mV for 10 s, before applying each test pulse. The holding potential was −60 mV. Top panels: by subtracting the aspecific currents remaining after Cs^+^ treatment (top panels), we obtained the pure inward component of *I*_IRK_. Note the large increase in membrane currents at *V*_rest_ values negative to *E*_k_, in both ‘Control’ and ‘Control–Cs^+^’ traces. Bottom panels: by subtracting the currents in the presence of WAY from the control currents, we isolated hERG components, which turned out to be negligible in healthy tissue.

Several types of ion channels have been described in gliomas (Bordey and Sontheimer, 1998; Bubien *et al*, 1999; Ranson and Sontheimer, 2001; Olsen and Sontheimer, 2004). As described in the Introduction, we sought IRK-type inwardly rectifying currents (*I*_IRK_), and hERG (*I*_hERG_), or hELK2 (*I*_hELK2_) currents. IRK is a strong inward rectifier and thus is easier to detect at a membrane potential (*V*_m_) more negative than *E*_k_. On the other hand, hERG (hereafter referred to as hERG1) and hELK2 channels are voltage-dependent outward rectifiers, structurally related to the *Shaker* family of K^+^ channels (Warmke and Ganetzky, 1994). However, they are easier to detect when current is flowing inward as well, since large ‘tail currents’ can be revealed on repolarisation after conditioning at *V*_m_⩾0 mV. During the conditioning step, hERG1 (or hELK2) activates and rapidly inactivates. On repolarisation, the inactivation is removed within milliseconds, revealing a large tail current which decays with a relatively slow kinetics, since the deactivation process is about two orders of magnitude slower than recovery from inactivation (for details on hERG1/hELK2 in cell lines and tumours, see Faravelli *et al*, 1996; Becchetti *et al*, 2002). To magnify the K^+^ currents at practical *V*_m_'s, we usually applied our stimuli in the presence of 40 mM external potassium (giving a Nernst potential around −30 mV). A typical example of our experimental procedure for measuring whole-cell currents in non-neoplastic tissue is illustrated in Figure 1. Negligible currents were observed at *V*_m_ more positive than *E*_K_, whereas robust inward currents appeared around −40 mV. Subsequent application of 1 *μ*M WAY (a specific inhibitor of hERG channels, when used at this concentration) had no effect, suggesting that the inward current does not contain any hERG component. Uncompensated capacitive transients and linear leak were subtracted by using Cs^+^ concentrations between 5 and 20 mM. The current/voltage relation obtained in this way is quasi-linear at *V*_m_ more negative than *E*_K_.

We tested five samples of healthy tissue. Four of them expressed measurable *I*_IRK_. On average, the percentage of cells showing *I*_IRK_ in each sample was 30±11%, with a current density of −22±4.4 pA pF^−1^, at −100 mV. The statistics for K^+^ currents in controls and gliomas are given in Table 2. No evidence was found for *I*_hERG_ or *I*_hELK2_ in healthy tissue. The distribution of *I*_IRK_ and the other K^+^ currents according to tumour grade is given in Figure 4 (white bars), along with the distribution of the corresponding transcripts (black bars). The comparison of the two sets of data is discussed below.

**Figure 2:**
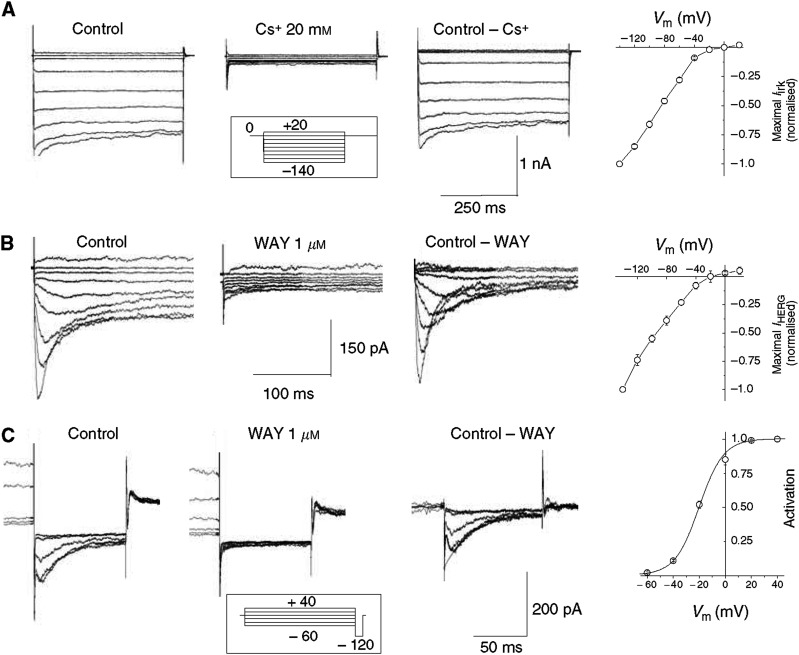
*I*_IRK_ and *I*_hERG_ in astrocytoma cells. (**A**) Representative whole-cell *I*_IRK_ traces, measured from glioma cells, by using the procedure described in Figure 1. The stimulation protocol is shown in the inset. The conditioning step at 0 mV lasted 10 s. Holding potential was −60 mV. *I*_IRK_ was isolated by subtracting the current recorded in the presence of Cs^+^ from the total current. In this astrocytoma case, no evidence for *I*_hERG_ was found. The panel on the right plots the average normalised peak currents as a function of the test potential, from four experiments. (**B**) Whole-cell *I*_hERG_ traces, from a different astrocytoma case, elicited by using the same stimulation protocol illustrated in panel A. *I*_hERG_ was isolated by subtracting the current obtained in the presence of 1 *μ*M WAY from the total current. The panel on the right plots the corresponding *I*/*V* curve. Data points are average normalised peak currents plotted as a function of the test potential. Currents were measured in five cells from the same case. (**C**) Whole-cell tail *I*_hERG_ at −120 mV, elicited from 3 s conditioning pulses from −60 to + 40 mV, as shown in the inset. Pure *I*_hERG_ was isolated by applying 1 *μ*M WAY. Peak tail currents were estimated by extrapolating the currents at −120 mV to the start of pulse (as indicated in the ‘Control–WAY’ panel). In this way, we corrected for the partial deactivation occurring before the fast recovery from inactivation is complete. Data points are average maximal tail current values at −120 mV, plotted as a function of the conditioning potential, measured in three experiments from the same glioma case.

Figure 2 (panel A) shows typical *I*_IRK_ from glioma cells. The *I*/*V* curve was generated by plotting the average normalised peak currents from four cells cultured from the same glioma case, as a function of test potential. Different samples gave similar results. The percentage of tumour cases expressing *I*_IRK_ was 50% in As, 57% in GBMs, 67% in OD/OAs and 100% in Eps (Figure 4). Among these, the average percentage of cells showing *I*_IRK_ was around 20–30%, irrespective of tumour grade, a value comparable to that observed in the controls. However, the corresponding current densities were significantly lower in GBM and OD/OA, compared to controls (Table 2).

*I*_hERG_ was isolated by applying the same stimulation protocol both in the absence and presence of 1 *μ*M WAY. The current recorded in the presence of the inhibitor was then subtracted from the total current, to reveal a WAY-sensitive component showing the typical kinetics of *I*_hERG_ (Figure 2, panel B). The corresponding *I*/*V* curve was generated from five cells cultured from the same glioma case. The *I*_hERG_ activation curve for glioma cells was obtained from tail currents at −120 mV, after 3 s conditioning steps from −60 to +40 mV. The duration of the conditioning steps is an acceptable compromise between the necessity of applying long conditioning steps for achieving steady-state conditions and the practical need for mantaining the duration of each experiment within reasonable limits to avoid cell damage (Schönherr *et al*, 1999). Typical current traces are shown in Figure 2 (panel C). Once again, *I*_hERG_ was isolated by applying WAY. The corresponding activation curve was obtained from three cells of the same glioma. For *I*_hERG_ also, different samples gave similar results. An alternative approach to distinguish *I*_IRK_ from *I*_hERG_ or *I*_hELK2_ is application of 100 *μ*M Cs^+^ or Ba^2+^. Such concentrations are ineffective on hERG/hELK2 channels (Arcangeli *et al*, 1995; Becchetti *et al*, 2002), but strongly inhibit IRK. The latter procedure is particularly useful to isolate *I*_IRK_ from *I*_hELK2_, which is insensitive to WAY. The isolation and the functional properties of *I*_hELK2_ expressed in human astrocytoma cells have been recently described by us (Becchetti *et al*, 2002) and will not be reproduced here. In general, there was no overlap of *I*_IRK_ and *I*_hERG_. In the samples that expressed both, most of the cells that we tested showed either the former or the latter. In GBM, in particular, among the cells expressing at least one K^+^ current type, 50% expressed *I*_IRK_, 42% expressed *I*_hERG_ and 8% expressed both. Finally, three out of 21 glioma cases showed *I*_hELK2_ that was expressed in about 22% of the tested cells, with a current density of −17.4±6 pA pF^−1^ and no overlap with *I*_hERG_ (Table 2).

### K^+^ channel transcript expression in primary human gliomas

**Figure 3:**
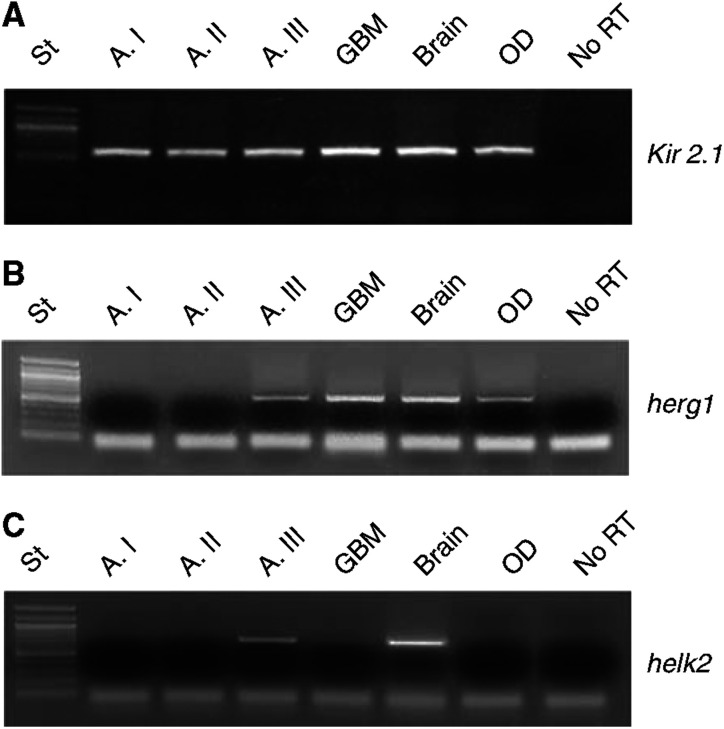
Reverse transcription–PCR of *Kir 2.1*, *herg1* and *helk2* transcripts in a representative pool of glioma samples. RNA extraction, RT and PCR amplification of the three transcripts was performed as reported in Materials and Methods. Only samples where a good amplification of the housekeeping gene *gapdh* (not shown) was achieved were processed for further analysis. DNA marker: 100 bp (New England Biolabs); the ‘no-RT’ lane contains a sample of RNA added to the PCR reaction mixture without previous RT. Lane labelled ‘Brain’ refers to total human brain RNA (see Materials and Methods). (**A**) Kir 2.1 transcript; (**B**) herg1 transcript; (**C**) helk2 transcript.

The electrophysiological data show that the ion channels under investigation are expressed on the plasma membrane and present usual properties. It was however necessary to complement this biophysical evidence with molecular data. mRNA and protein expression patterns do not always fully overlap; thus, a study based on mere electrophysiological analysis could underestimate the importance and utility of these marker gene. In addition, RT–PCR allows the molecular identification of the ion channels responsible for the ion currents recorded by patch-clamp investigation. To address these issues, we performed an RT–PCR survey of the most likely candidate genes: *Kir 2.1*, a molecular component of *I*_IRK_, known to be highly expressed in cortical astrocytes (Olsen and Sontheimer, 2004), *herg1*, a molecular component of *I*_hERG_, the member of the *herg* family most frequently expressed by tumour cells (Crociani *et al*, 2003) and *helk2*. Representative examples of the PCR-amplified transcripts are reported in Figure 3.

**Figure 4:**
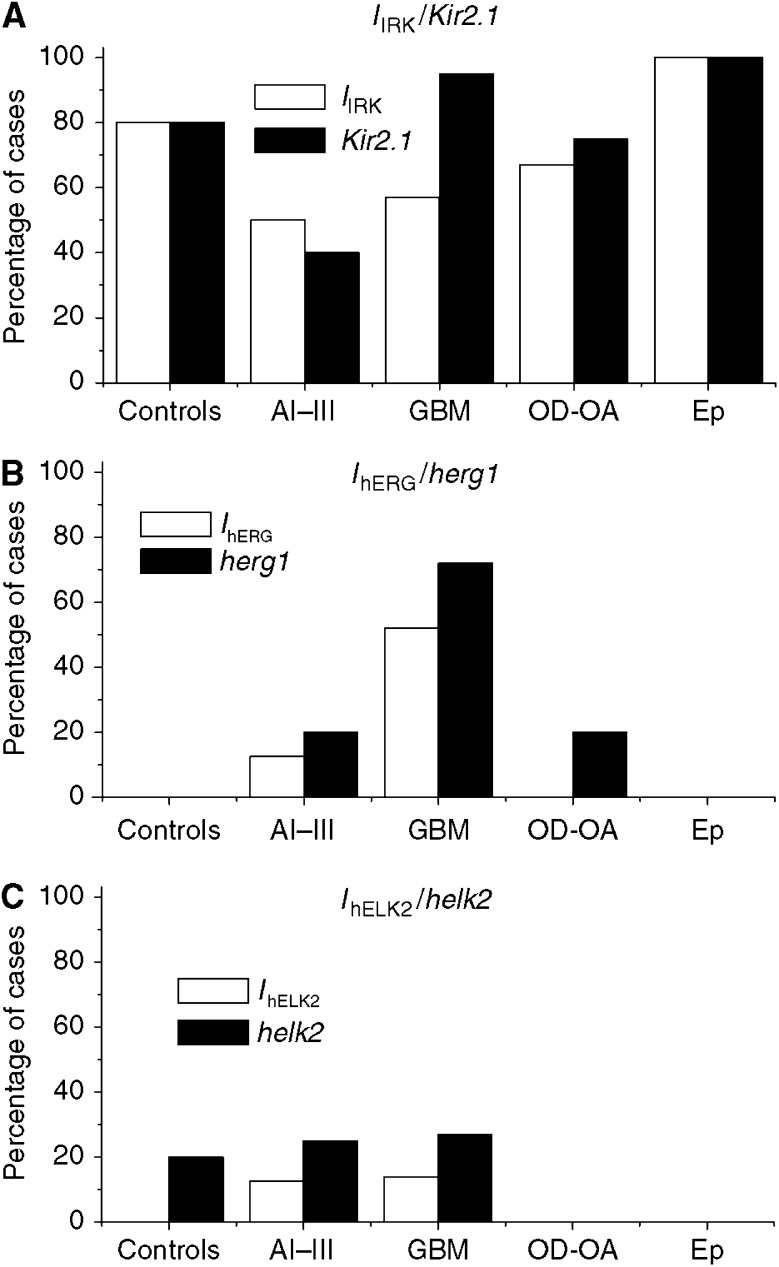
Correlation of the expression of *I*_IRK_, *I*_hERG_, *I*_hELK2_ and the corresponding transcripts with tumour grade, in primary gliomas. Bars represent the percentage of cases expressing the indicated K^+^ current (white bars), or the corresponding transcript (black bars), among the indicated tumour classes. Data were obtained by patch-clamp for K^+^ currents and RT–PCR for transcript expression (see text). Panels A–C show the results obtained for *I*_IRK_/*Kir 2.1*, *I*_hERG_/*herg1* and *I*_hELK2_/*helk2*, respectively. Controls: normal cases; AI–III: astrocytoma WHO grade I–III; GBM: glioblastoma multiforme, WHO grade IV; OD: oligodendroglioma; OA: oligoastrocytoma; Ep: ependimoma.

Figure 4 summarises the expression of the three potassium channel-encoding genes according to grade (black bars). *Kir 2.1* transcript was expressed in 80% of the controls, 40% of the As, 90% of GBMs, 70% of OD/OA and 100% of the Eps, with no obvious relation to tumour grade (Figure 4, panel A). On the other hand, *herg1* transcript showed a considerably lower overall expression, except that in high-grade gliomas (Figure 4, panel B). Finally, the *helk2* transcript was detected in a relatively low percentage of cases, with no clear relation to tumour types (Figure 4, panel C).

### Comparison of K^+^ current and transcript expression

Results obtained with patch-clamp and RT-PCR were, generally, in good agreement. IRK was expressed in the vast majority of normal and glioma cases examined, irrespective of the histotype and WHO grade. This suggests that this channel type is unlikely to play a critical role in gliomagenesis or tumour progression. IRK expression may actually be inversely related to neoplastic transformation. In fact, in GBMs, *Kir 2.1* was detected in 95% of the cases, whereas a detectable *I*_IRK_ was only found in 57% of the GBM tested, with a current density significantly lower than in the controls (Table 2). Our results are consistent with those reported by Olsen and Sontheimer (2004), who found a reduction of *I*_IRK_ in glioma cell lines, due to diminished translation and mislocalisation of the Kir 2.1 protein. Our data suggest that such a diminished translation (or mislocalisation) of IRK-type channels occurs not only in high-grade glioma cell lines but also in tumour-derived GBM cells. Therefore, downregulation of IRK may be seen as a distinctive feature of all the cycling and immature cells of glial origin, most prominent in highly malignant cells.

Our results on hERG1 show that mRNA expression and *I*_HERG_ detection showed the same pattern, with increased expression in more malignant gliomas. Our observations also suggest that, unlike *Kir 2.1*, hERG1, when present at the mRNA level, is probably correctly expressed in the membrane, although we lack a comparison with *I*_hERG_ density in the controls (Table 2).

The results on hELK2 seem also consistent. The only discrepancy was that the transcript expression in the control sample was not accompanied by *I*_hELK2_ detection. This may be simply due to the limited sampling possible with patch-clamp technique.

Thus, similarly to what we reported on colorectal cancers, hERG1 channel expression was higher in tumour glia and correlated with an aggressive phenotype.

### IHC for hERG1

**Figure 5:**
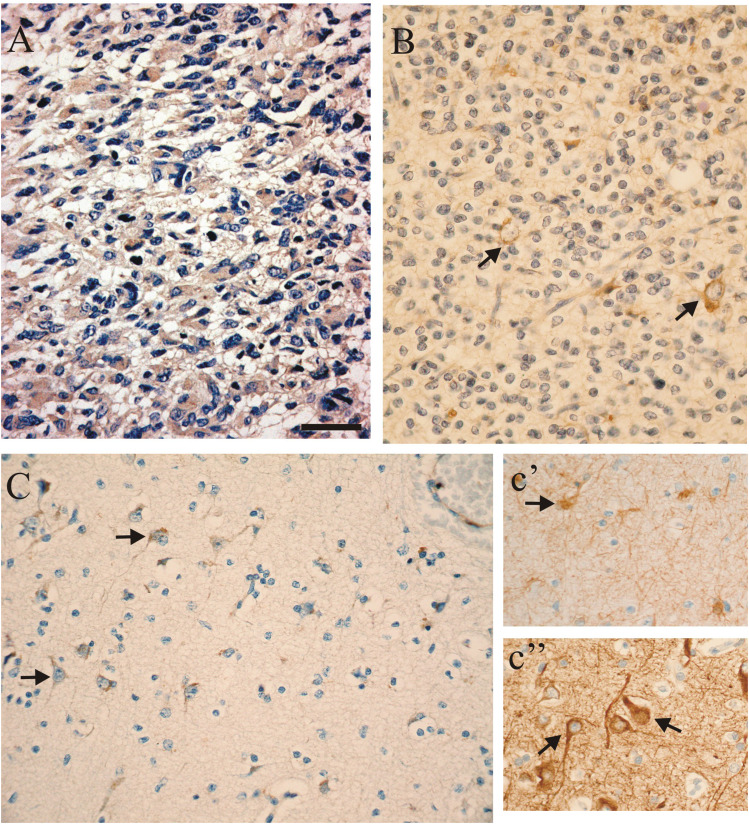
Immunohistochemical detection of hERG1 protein in gliomas and normal brain tissue. Sample preparation, antibodies used and IHC procedures are reported in Materials and Methods. (**A**) Immunohistochemistry with anti-hERG1 antibodies in a GBM; (**B**) IHC with anti-hERG1 antibodies in an OD; note the lack of staining in neoplastic oligodendrocytes compared to the positive staining of neurons entrapped in the neoplastic lesion; (**C**) IHC of a normal brain sample using anti-hERG1 antibodies; c′=GFAP staining (note the positive astrocytes); c″=NF staining (note the positive neurons). Bar=50 *μ*m.

To exclude the possibility that hERG1 expression was an artefact due to culturing conditions, we have tested whether the channel protein was expressed in primary GBM *in situ*, by using anti-hERG1 antibodies on those cases where histological sections were available. The antibodies had been previously tested in human colorectal cancers (Lastraioli *et al*, 2004). Representative photographs are shown in Figure 5. Panel A shows the pattern of hERG1 protein expression in a GBM (case no. 10 in Table 1), in which both *herg1* gene expression and *I*_hERG_ had been detected. The localisation of hERG1 in the cytoplasm of neoplastic glial cells is apparent and evidenced as a brown staining. Panel B shows an OD case (no. 37 in Table 1) that had neither *herg1* gene expression nor *I*_hERG_ and, consistently, no immunoreaction. It is interesting to notice that the few neurons entrapped in the tumour lesion, which are easily recognisable by their nuclei, showed a positive cytoplasmic staining for the hERG1 protein. The same pattern of hERG1 expression (neurons positive and glial cells negative) also occurs in normal brain tissue. This can be seen in panel C, which illustrates an area of normal tissue from outside of the tumour boundaries. Neurons were identified by staining with an NF antibody (c″) and were also positive for hERG1, while astrocytes (GFAP-positive cells in inset c′) showed no sign of hERG1 expression.

The immunohistochemical data support our conclusion that: (1) hERG1 channel is more frequently expressed in human gliomas; (2) the frequency of channel expression correlates with tumour grade; (3) the channel is absent in normal human glial cells. We suggest that *herg1* gene and the corresponding protein can be conceivably employed as markers of tumour progression in glial tumours, in analogy with what we have reported in colorectal cancers.

### *I*_hERG_ inhibition reduces VEGF secretion in glioblastoma cell lines

We noticed that the few GBMs negative for hERG1 expression were secondary (Sec) cases, arising from previous As of lower grade. Interestingly, primary GBMs can be distinguished from secondary GBMs for their expression of angiogenesis-related genes (Godard *et al*, 2003). We asked whether the two findings may be linked and we made the hypothesis that HERG1 channel's activity is involved in the regulation of angiogenesis in gliomas, possibly by affecting the secretion of angiogenic factors, such as VEGF.

To build on the correlative evidence, we determined the amount of VEGF secreted by the GBM cell lines U138 and A172, after blocking *I*_hERG_. Preliminarily, we examined the expression of *Kir 2.1*, *herg1*, *helk2* and *heag* transcripts in both cell lines. *hEAG* was included in the screen because it is expressed in human primary gliomas (Patt *et al*, 2004), and because hERG channel inhibitors also block hEAG currents at high concentrations (Gessner and Heinemann, 2003; Gessner *et al*, 2004).

**Figure 6:**
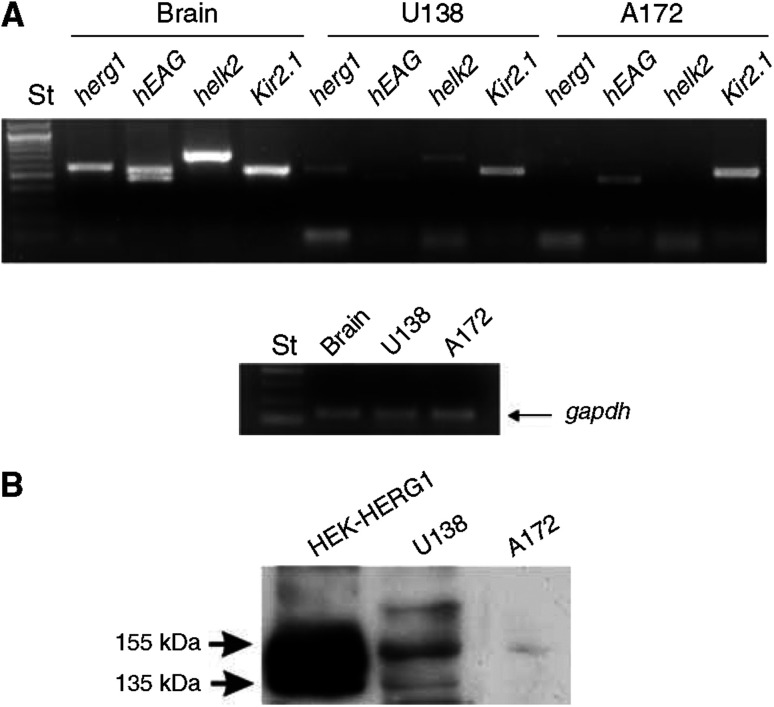
K^+^ channel expression in glioma cell lines. (**A**) Expression of K^+^ channel transcripts. Reverse transcription–PCR was performed on RNA extracted from U138 and A172 cells. Brain cDNA was used as a positive control. Note that in the sample ‘brain’ two PCR bands relative to *heag* were detected, the lower corresponding to the so-called *heag1* (NM 002238), the upper corresponding to the longer isoform of the gene (known as *heagb* or transcript variant 1, NM 172362). The two cell lines expressed only the lower band. Lower panel, RT–PCR of the control gene *gapdh*, from the samples above. (**B**) Western blotting with anti-hERG1 antibody of *herg1*-transfected HEK 293 cells (HEK-hERG1), U138 cells and A172 cells.

It emerged that both U138 and A172 cells expressed *Kir 2.1* and *hEAG* transcripts, but only U138 cells expressed *herg1* as well as *helk2* (Figure 6A).

The finding that *herg1* is expressed in U138 cells only was confirmed at the protein level. Western blotting performed on both cell lines and on hERG1-transfected HEK 293 cells, for control, confirmed that only U138 cells expressed the hERG1 protein (Figure 6B). The mean *V*_rest_ of U138 cells was −18 mV, which is similar to the values reported for hERG1-expressing tumour cells (Arcangeli *et al*, 1995; Schönherr *et al*, 1999). Moreover, WAY induced a dose-dependent *V*_rest_ depolarisation (Table 3), similarly to that we normally observed in HEK-hERG1 cells. Conversely, A172 showed a *V*_rest_ value around −23 mV that was insensitive to WAY (Table 3).

**Figure 7:**
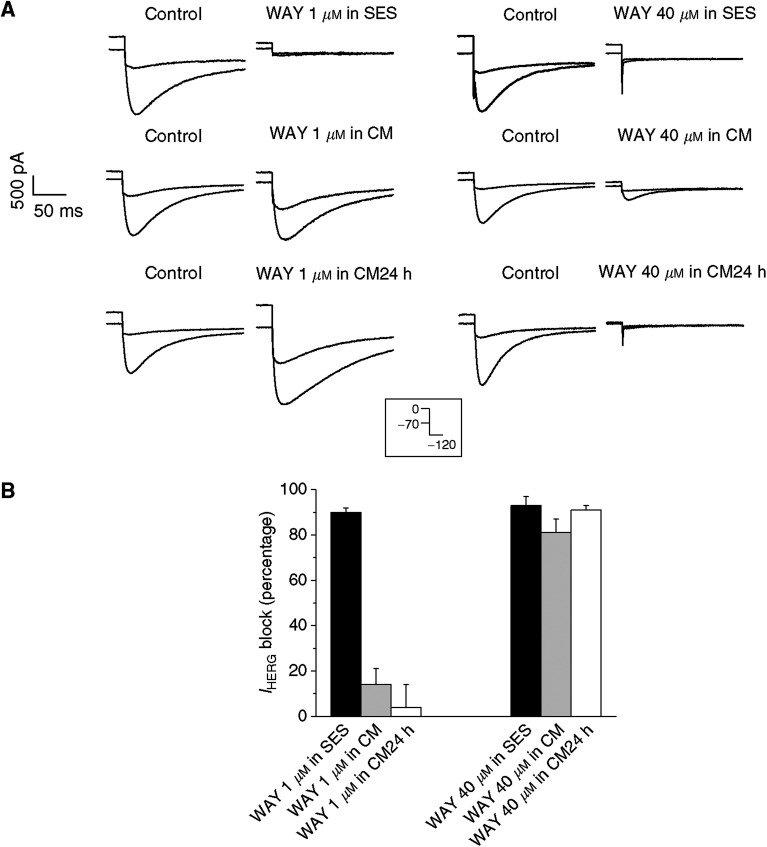
WAY inhibition of *I*_hERG_ in different experimental conditions. Whole-cell *I*_hERG_ traces were studied as tail currents at −120 mV (for 80 ms), preceded by 10 s conditioning steps at 0 and −70 mV, as shown in the inset. Currents were measured in the absence (control) or presence of the indicated concentration of WAY. (**A**) Effects of 1 and 40 *μ*M WAY diluted in (1) SES (upper panels); (2) culture medium incubated 15 min at 37°C, 5% CO_2_ (CM, middle panels); (3) CM incubated 24 h in the dishes containing the cells, at 37°C and 5% CO_2_ (CM 24 h, lower panels). (**B**) Percentage of *I*_HERG_ block, calculated as explained in Materials and Methods, in the experimental conditions indicated in (**A**).

Furthermore, we determined the dose dependence of WAY inhibition on *I*_hERG_, in the experimental conditions adopted for measuring VEGF secretion. In fact, the efficacy of hERG blockers might be altered by protein binding of the drug (Webster *et al*, 2001), or perhaps progressive degradation during long incubation. Thus, we tested the efficacy of WAY on *I*_hERG_ after diluting the drug: (1) in SES, normally used for our patch-clamp experiments; (2) in the culture medium used for growing glioma cells during secretion experiments (CM); (3) in CM medium preincubated for 24 h in the same dishes containing the cells (CM 24 h). These experiments were carried out on our HEK-hERG1 stable cell line. *I*_hERG_ was studied as tail current at −120 mV, after conditioning for 10 s at 0 mV (to produce maximal activation), and, subsequently, at −70 mV (to produce strong current deactivation, as further control that we were specifically measuring *I*_hERG_). As reported in Figure 7, 1 *μ*M WAY produced almost complete inhibition only when diluted in SES (average percentage *I*_hERG_ block was 90±2%). On the other hand, the effect of 1 *μ*M WAY was drastically reduced after dilution in CM or CM 24 h. On the contrary, 40 *μ*M WAY was strongly effective in all the experimental conditions that we have applied.

The above preliminary tests suggest that U138 and A172 cells are suitable models for the study of the dependence of proangiogenic factor secretion on hERG1 channel block. Since U138 cells express functional hERG1 channels and A172 cells do not, the latter can be used as negative controls when studying the sensitivity of VEGF secretion to WAY treatment. In addition, high concentrations of WAY must be used when cells are tested after 24 h incubation in culture medium.

**Figure 8:**
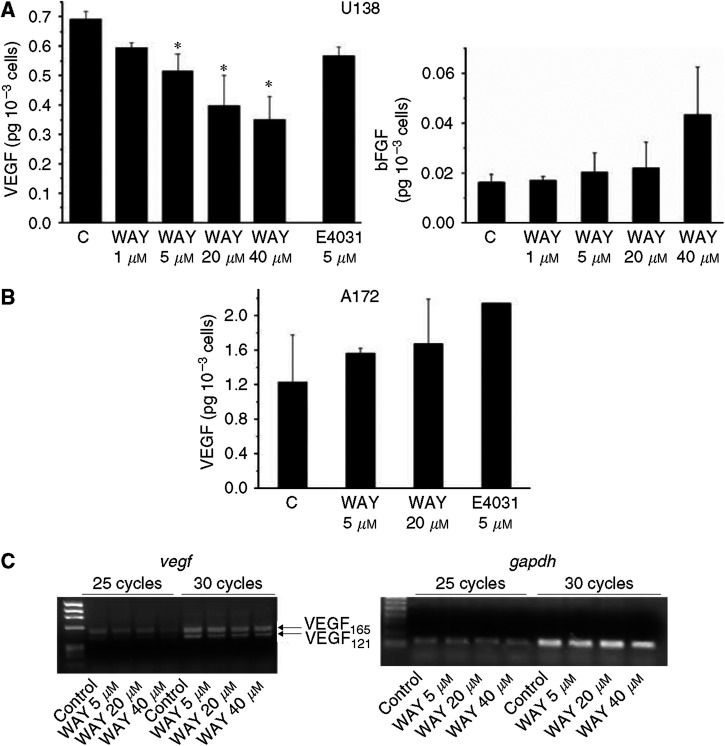
Vascular endothelial growth factor secretion in U138 and A172 glioma cell lines. (**A**) Effects of WAY and E4031 on VEGF secretion in U138 cells. The amount of VEGF secreted into the medium was determined with an ELISA performed in triplicate (see Materials and Methods) after cells were incubated for 24 h in serum-free medium. The kit used allows detection of both VEGF_121_ and VEGF_165_. WAY and E4031 were added at time zero, at the final concentrations indicated below. Values are expressed as pg of secreted VEGF/10^3^ cells during 24 h incubation. The number of cells per well was the following: Control: 5.98±0.59 × 10^5^ (*n*=11); WAY 1 *μ*M: 6.31±0.49 × 10^5^ (*n*=3); WAY 5 *μ*M: 5.16±1.12 × 10^5^ (*n*=5); WAY 20 *μ*M: 5.09±1.08 × 10^5^ (*n*=4); E4031 5 *μ*M: 7.58±0.5 × 10^5^ (*n*=3). Data reported are means±s.e.m. of three to five separate experiments, each carried out on triplicate samples. ^*^Significantly different from the control (Student's *t*-test for paired samples): WAY 5 *μ*M: *P*=0.03; WAY 20 *μ*M: *P*=0.01; WAY 40 *μ*M: *P*=0.003. Inset: bFGF secretion in U138 cells in the absence or presence of WAY. The same supernatants as those used for VEGF measurement in panel A were used for the detection of the secreted bFGF, using an ELISA (see Materials and Methods). Note the different amount of secreted bFGF as compared to VEGF. (**B**) Effect of WAY and E4031 on VEGF secretion in A172 cells. Vascular endothelial growth factor was quantified as reported in (**A**). WAY (5 and 20 *μ*M, final concentrations) and E4031 (20 *μ*M, final concentration) were added at time zero. The number of cells per well was the following: Control: 2.21±0.03 × 10^5^ (*n*=3); WAY 5 *μ*M: 2.18±0.15 × 10^5^ (*n*=2); WAY 20 *μ*M: 2.18±0.04 × 10^5^ (*n*=2); E4031 5 *μ*M: 2.06 × 10^5^ (*n*=1). Data reported are means±s.e.m. of two to three separate experiments, each carried out on triplicate samples. It is worth pointing out that the difference in the amount of basal VEGF secretion between U138 and A172 cells can be attributed to the different cell size. (**C**) Semiquantitative analysis of *vegf* mRNA expression after WAY addition. *Vegf* expression was determined after 24 h of cell incubation in the absence or presence of increasing concentrations of WAY (5, 20 and 40 *μ*M). PCR conditions were adjusted in order to determine the number of cycles corresponding to the exponential phase of amplification, when semiquantitative analysis is possible. Densitometry was performed with Scion Image software by comparing the intensity of *vegf* (25 cycles) and *gapdh* (20 cycles) PCR products. *Vegf* expression was inhibited by approximately 30% by treatment with 40 *μ*M WAY.

Finally, we studied the effect of blocking *I*_hERG_ on VEGF secretion by the above GBM cell lines. Secretion was measured after a 24 h incubation of U138 and A172 cells in the appropriate culture medium (see Materials and Methods), in the presence of increasing concentrations of WAY (Figure 8). U138 cells secreted VEGF into the culture medium and this secretion was inhibited by WAY in a dose-dependent manner, with a roughly 50% inhibition at 40 *μ*M (panel A). Conversely, WAY did not inhibit VEGF secretion in A172 cells (panel B). Our data strongly suggest that the effect of WAY on VEGF secretion of glioma cells is due to specific inhibition of *I*_hERG_, for two reasons. First, secretion is only inhibited in glioma cell lines that express functional hERG1 channels. Second, the dose–response of VEGF secretion to WAY parallels the response of *I*_hERG_ to WAY in culture conditions (Figure 7). This conclusion is further supported by the observation that treatment with 5 *μ*M E4031, another specific hERG1 channel blocker, produced a partial inhibition of VEGF secretion only from U138 cells (compared to the null effect of E4031 on A172 cells, reported in panel B). It is worth noting that the effects of hERG channel blockers in this model were not accompanied by any effect on cell proliferation (see legend to Figure 8), such as that occurring in other hERG1-positive cell lines (Arcangeli, 2005; Arcangeli and Becchetti, 2006).

The effect of WAY was specific to VEGF secretion; in addition to VEGF, bFGF is also secreted by U138 cells, although in lower amounts; however, its secretion was insensitive to WAY (inset to panel A). The apparent specificity of WAY to VEGF secretion led us to investigate the possible mechanisms of this inhibition. Since the regulation of VEGF secretion has been attributed to the modulation of transcription in multiple cell types including glioma cells (Steiner *et al*, 2004), a semiquantitative RT–PCR of *vegf* (corresponding to VEGF_121_ and VEGF_165_) transcripts in U138 cells was performed under control conditions and in the presence of increasing concentrations of WAY (Figure 8, panel C). *gapdh* was used as a reference for semiquantitative analysis. A dose-dependent inhibition of transcription of both VEGF_165_ and VEGF_121_ was observed in U138 cells treated with WAY, with a 30% reduction when using the highest concentration (40 *μ*M) of the hERG inhibitor.

We propose that hERG1 channel activity, and the channel's overexpression in highly malignant tumours, contributes to regulating the production/secretion of angiogenic factors from glial tumour cells, in particular of VEGF, and that this regulation may rely on the modulation of *vegf* transcription levels.

## CONCLUDING REMARKS

The data reported in this paper lead us to conclude that *herg1* is expressed, and the corresponding *I*_hERG_ is present, in glial tumours, especially in high-grade astrocytic tumour, namely GBM. In contrast, *I*_hERG_ and the corresponding gene turned out not to be significantly expressed in the controls, that is, normal human astrocytes, either in culture or in histological sections.

The expression of the *herg1* gene in human glial tumours has also been recently reported by Patt *et al* (2004). These authors, however, found that the transcript expression was lower in high-grade and higher in low-grade gliomas, especially in the control. The discrepancy between these data and ours may be traced back to the fact that Patt and co-workers tested *herg1* transcript from RNA extracted from the whole tissue. Since hERG1 channels are known to be expressed in neurons throughout the mammalian brain (Saganich *et al*, 2001; Polvani *et al*, 2003; see Figure 3), it is not surprising that a brain sample shows relatively strong hERG1 expression. To overcome this problem, we have compared acutely established normal astrocytes and glioma cell cultures, mostly expressing the glial phenotype (as evidenced by the GFAP staining).

The increased hERG1 expression in high-grade gliomas may imply that hERG1 expression correlates with tumour progression. In addition, we found little overlap of *I*_IRK_ and *I*_hERG_ in GBM, which usually had either the former or the latter. This suggests that the carcinogenic process may select for a malignant population which upregulates hERG1 and downregulates the expression of IRK onto the cell membrane. This would be analogous to what happens in colorectal cancers (Lastraioli *et al*, 2004), where the *herg1* gene and hERG1 protein expression mark metastatic Dukes' D cancers. A definitive conclusion, however, is more difficult to reach for all gliomas, because the majority of surgical specimens represent high-grade GBM.

While in many other human cancers hERG1 activity contributes to the regulation of either cell proliferation or invasion, in GBM cells the activity of hERG1 channels seems to be related to a different aspect of tumour cell progression, that is, neoangiogenesis. This would occur through the modulation of VEGF secretion, which was inhibited when hERG1 channel activity was specifically blocked. Our data also suggest that the mechanism whereby hERG1 channels stimulate secretion depends on a modulation at the mRNA level, similarly to what we have previously reported in leukaemia cells for the calcitonin receptor (Hofmann *et al*, 2001).

Providing further evidence confirming that hERG1 exerts proangiogenic effects will require studies carried out in conditions of hypoxia: poor blood supply and reduced O_2_ tension are often associated with malignant gliomas. In these conditions, hERG1 channel activity is upregulated (Fontana *et al*, 2001) and VEGF secretion is stimulated as a result of activation of the HIF pathway (Semenza, 2004).

Finally, does the regulatory effect of hERG1 depend on K^+^ current flow or is it triggered by a conformational change of the channel protein independently from the consequent biophysical effects? Since the ion selectivity and the voltage dependence of hERG1 and hELK2 are very similar, if the signaling pathways that lead to VEGF secretion were triggered by a biophysical effect, we would expect that individual gliomas selected either hERG1 or hELK2 expression, indifferently. Our data show instead that hERG1 largely prevails. Moreover, data on U138 cells suggest that *helk2* does not substitute for *herg1*. Therefore, it seems that secretion is stimulated by unknown regulatory effects triggered by hERG1 (and not by other K^+^ channels), although ion currents may have an effect of their own. In other contexts, hERG1 channels are known to interact with other membrane proteins with signalling function. We have recently shown that hERG1 is physically linked to beta1 integrins and thereby modulate adhesion-dependent signaling (Cherubini *et al*, 2005).

Nonetheless, our observations lend further support to the idea that hERG1 channels should be included among the molecular markers of gliomagenesis and possible targets for novel antitumour therapies.

## Figures and Tables

**Table 1:** Clinical–pathological data

**Histopathological diagnosis**	**WHO grade**	**Sample no.**	**Sex**	**Age**	**Location of tumour**
A	I	3	M	21	Chiasma
A	I	7	F	12	Right parietal
A	II–III	16	F	32	Left frontoinsular
A	II–III	17	F	50	Right frontotemporal
A	II–III	29	M	38	Left temporal
A (gemistocytic)	III	20	F	51	Left temporoparietal
A (anaplastic)	III	26	F	51	Mesial temporal
A (anaplastic)	III	30	F	60	Right frontolateral
GBM	IV	1	M	57	Right parietal
GBM	IV	2	F	72	Right frontal
GBM	IV	8	F	69	Left occipital
GBM	IV	10	F	75	Right parietoccipital
GBM	IV	11	M	44	Right frontoparietal
GBM (Sec)	IV	12	M	ND	Parietal
GBM (Gliosarcoma)	IV	13	M	50	Right occipital
GBM (Sec)	IV	14	F	46	Right frontal
GBM	IV	15	M	67	Right parasagittal
GBM	IV	5	F	67	Right frontoccipital
GBM	IV	6	M	58	Right temporoccipital
GBM	IV	18	F	53	White matter and thalamus
GBM	IV	19	M	47	Left temporal
GBM	IV	22	F	78	Left temporoparietal
GBM (Sec)	IV	23	F	37	Left frontoinsular
GBM	IV	24	M	37	Right temporal
GBM (Sec)	IV	28	M	31	Left frontal
GBM	IV	31	M	45	Left temporoinsular
GBM	IV	32	F	59	Left frontal
GBM	IV	33	M	53	Left frontotemporal
GBM	IV	34	F	68	Left parietal
GBM	IV	36	M	61	Right temporoccipital
GBM	IV	38	M	45	Left occipital
GBM	IV	39	M	69	Right parietal
GBM	IV	40	F	82	Left parietal
GBM	IV	41	F	74	Left fronto-insular
OD	II	9	M	70	Right frontotemporal
OD	II	21	F	74	Right frontal
OD	II	37	M	50	Left frontotemporal
OA	II–III	27	M	47	Left parietoccipital
OA	III	25	F	66	Left temporal
Ep	II	4	F	26	IV ventricle
Ep	III	35	M	33	IV ventricle

A=astrocytoma; GBM=glioblastoma multiformis; OD=oligodendroglioma; OA=oligoastrocytoma; Ep=ependimoma; Sec=Secondary; M=male; F=female.

**Table 2:** Statistics of *I*_IRK_ and *I*_hERG_ in controls and glioma cases

**Grade**	***N* cases (*N* cells)**	**Average current expression (%)**	**Current density (pA pF^−1^)**
*I*_*IRK*_			
Controls	4 (42)	30±11	−22.0±4.4 (11)
AI–III	3 (31)	22±14	−11.3±3.7 (6)
GBM	12 (153)	28±4	−12.4±1.7 (40)^*^
OD/OA	2 (16)	29	−4.4±0.7 (4)^*^
			
*I*_*hERG*_			
Controls	0	NA	NA
AI–III	1 (6)	17	ND
GBM	11 (108)	35±8	−7.84±1 (24)
OD/OA	0	NA	NA
			
*I*_*hELK2*_			
Controls	0	NA	NA
AI–III	1 (4)	25	ND
GBM	3 (45)	22±3	−17.4±6 (9)
OD/OA	0	NA	NA

*N* cases=number of cases in which the indicated K^+^ current was detected; average current expression=the fraction of cells showing the indicated current was calculated for each case and the results thus obtained were averaged; current density=average current density at −100 mV; ND=not determined; NA=not applicable. Cell count is reported in parentheses. ^*^Significantly different from the controls (0.01<*P*<0.05).

**Table 3:** Effect of WAY on *V*_rest_ of U138 and A172 glioma cells

**Cell line**	***V*_rest_ (mV) Control conditions**	***V*_rest_ (mV) (1 *μ*M WAY)**	***V*_rest_ (mV) (40 *μ*M WAY)**
U138	−18±3 (7)	−12±2 (7)	−6±2 (7)
A172	−22±2 (7)	−24±1 (7)	−22±2 (7)

*V*_rest_ and its variation after application of 1 and 40 *μ*M WAY were determined as reported in Materials and Methods. Values are means±s.e.m.; cell count is reported in parentheses.
